# Influence of composite resin core buildup translucency on the accuracy of an anterior CAD-CAM bridge fabricated with a digital impression

**DOI:** 10.4317/jced.62249

**Published:** 2025-04-01

**Authors:** Nguyen Chi Tran, Nhat Dinh Minh Nguyen, Nam Cong Nhat Huynh, Trang Thi Ngoc Tran, Hung Trong Hoang, Ding Han Wang, Ming Lun Hsu

**Affiliations:** 1 Faculty of Dentistry, University of Medicine and Pharmacy at Ho Chi Minh City, Ho Chi Minh City, 749000, Vietnam; 2 Nikkori Dental Clinic, Ho Chi Minh City, 749000, Vietnam; 3 School of Dentistry, National Yang Ming Chiao Tung University, Taipei, 112304, Taiwan

## Abstract

**Background:**

Composite resin build-up translucency affects the accuracy of digital impressions generated by an intraoral scanning system (IOS). Here, we evaluated the influence of composite core translucency on the accuracy of a CAD-CAM bridge (Fixed Partial Denture) using an intraoral scanner.

**Material and Methods:**

We investigated the accuracy (the trueness and precision) of 2 different composites (EverX Flow-EX and G-aenial Universal Injectable A3) for core build up in 3-unit CAD/CAM bridge on anterior teeth using an intra-oral scanner (Trios 3, 3Shape) and injectable technique. The fitting of crown within the clinical acceptable threshold of final restoration was also confirmed by CBCT superimposition.

**Results:**

The results illustrated that composite with high translucency (A3) expressed lower trueness value than one with low translucency. With a clinically acceptable threshold<50μm, the percentage of points over the threshold was lower in composite group with low translucency (EX). CAD/CAM restorations on high translucency composite-reconstructed abutments showed a poor fit compared with the low translucency group on both abutments.

**Conclusions:**

The use of low translucency reconstructive materials helps to reduce the errors of IOS, and at the same time, appropriate compensation should be used when designing restorations to provide the most accurate results. Clinical Significance: • Composite with high translucency (A3) expressed lower trueness value than one with low translucency (EX). • With a clinically acceptable threshold<50μm, the percentage of points overcoming the threshold was lower in composite group with low translucency (EX). • Appropriate compensation should be applied when designing CAD/CAM restoration to achieve the best results.

** Key words:**Accuracy, intraoral-scanner, digital dentistry, resin composite, bridge prosthesis, CAD/CAM.

## Introduction

Francois Duret introduced CAD/CAM to dentistry (Computer-Aided Design/Computer-Aided Manufacturing) in 1973 under the name “Empreinte Optique” (Optical Impression). He then patented it as a digital device in 1984 (1). Since then, digital impressions have been widely used by dental professionals. Today, there are many intraoral-scanner (IOS) systems available on the market. The accuracy of digital impressions is crucial for successful CAD/CAM restorations. According to ISO 5725 standards, accuracy includes both “trueness” and “precision”. From the ISO standard: “Trueness” refers to the closeness of agreement between the arithmetic means of a large number of test results and the true or accepted reference value. “Precision” refers to the closeness of agreement between test results. Several factors can affect the accuracy of IOS scan data, including the scanning span, lighting conditions, operator experience, and tooth loss conditions. Restorative materials can also impact IOS image accuracy (2-5).

A 2015 study discovered that the dimensions of metal, ceramic, and composite surfaces’ images reconstructed from IOS scanning can be altered, which can lead to the failure of the final restoration if proper compensation is not applied. Among restorative materials, only composite creates a scale reduction of optical impression compared to the actual size of the abutment, which can result in irreparable fitting errors of final restorations (6). In our previous study, it was found that composite translucency affects the accuracy of optical impressions in core build-up restoration of single anterior incisor models (7). A higher translucency composite results in a less accurate digital impression. While numerous studies have explored the accuracy of monolithic ceramic restorations, none of them specifically address multi-unit restorations in cases of missing teeth (8,9). This gap in research warrants further investigation to enhance our understanding of the accuracy and performance of multi-unit restorations. We extended our assessment on the in-vitro situation of abutment cental incisor left loss with lateral incisor left, central incisor right abutments reconstructed by composite with different translucency, especially on the final restoration’s fitting. Our findings can help guide the selection of composite restorative materials for optimal clinical results.

## Material and Methods

1. Study design

**Figure 1:**
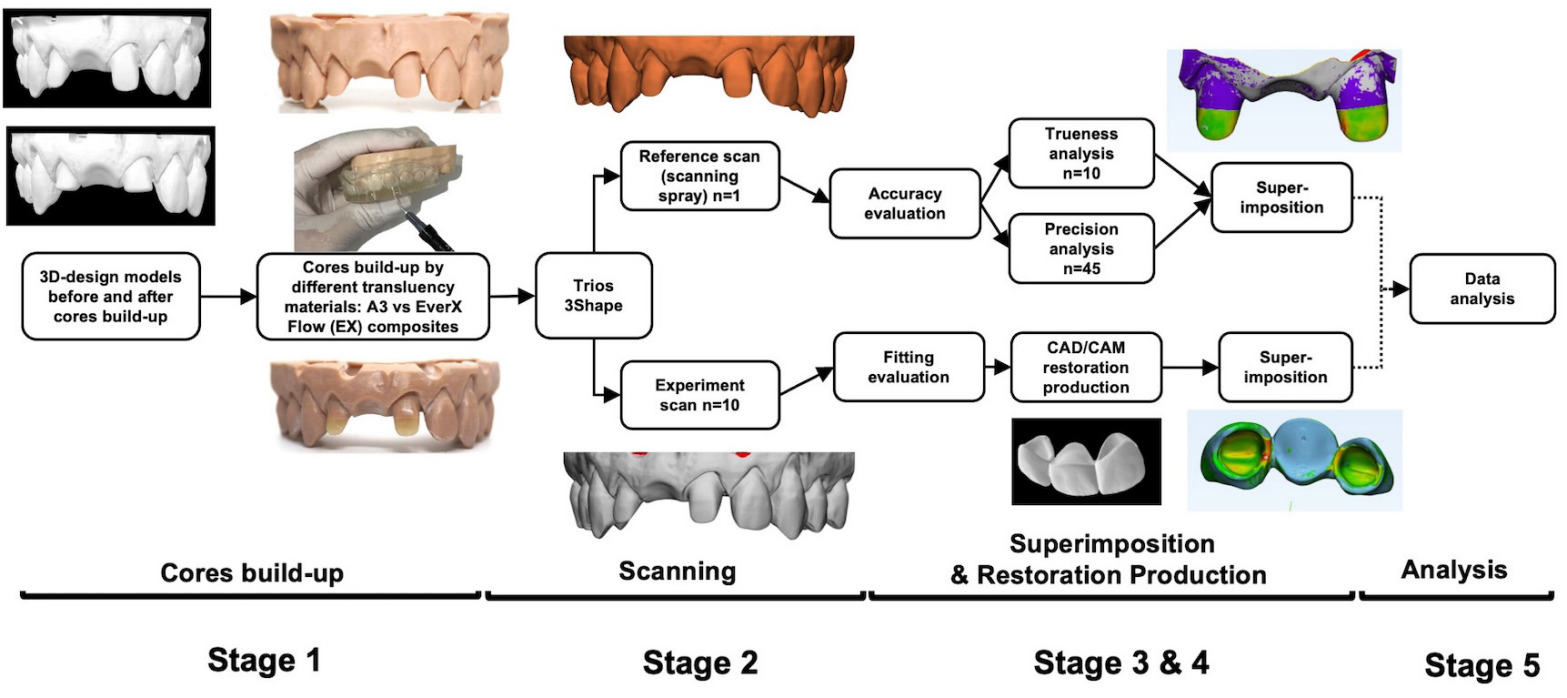
Conceptual framework of the study. The workflow was divided into 5 main stages: 1. core build-up, 2. scanning, 3. image superimpose, 4. CAD/CAM restoration production and 5. analysis.

In this study, we investigated the effect of two different composites used for core build up in the fabrication of a 3-unit CAD/CAM bridge on anterior teeth and detected the fitting error within the clinical acceptable threshold of final restoration. Fig. 1 shows the conceptual framework of the study. The workflow was divided into 5 main stages: 1. Core build-up, 2. Scanning, 3. Image superimposition, 4. CAD/CAM restoration production and 5. Analysis of accuracy.

2. *In-vitro* core build-up design 

Based on our earlier findings on translucency and accuracy (7), two types of composite with varying degrees of translucency were chosen for the current study. The following was the order of translucency level, from high to low: EverX Flow-EX (LOT 1912021, GC) and G-aenial Universal Injectable A3 (LOT 1812141). These composites could be used for abutment restoration core build-up. A proprietary maxillary model was created using Mimics Research software (version 21.0, Materialize N.V.,) with left central and right lateral incisors as abutments and the right central incisor as pontic. The heights of the abutments are about 2-3mm. The plan was to use resin composite to rebuild these abutments before the fabrication of a CAD/CAM bridge to restore them. The models and dies were 3D-printed (Sol 3D, Ackuretta Tech).

A clear polyvinyl siloxane index of the final shaped abutment was fabricated (Exaclear, GC, LOT 2005111) to carry out the composite injection and guarantee that the composite core build-up abutments had the same shapes. The following is how the core build-up process was conducted:

-Teflon tape was used to isolate two adjacent teeth canine right and lateral incisor left

-A hole bored at the incisal edges allowed the composites to be injected through the clear index, which was securely fastened to the model. The stages for bonding and etching were omitted.

-After photopolymerization, the abutments were polished.

3. Scanning procedure

Using an intra-oral scanner (Trios 3, 3Shape), each model with core built-up abutments was scanned ten times for the experimental scan data. The scanning procedure adhered to the manufacturer’s recommendation. Then the abutments were powder-coated, and the model was scanned to obtain the reference data. All the scan files were exported and stored in the standard tessellation language (STL) format.

4. Superimposition procedure

**Figure 2:**
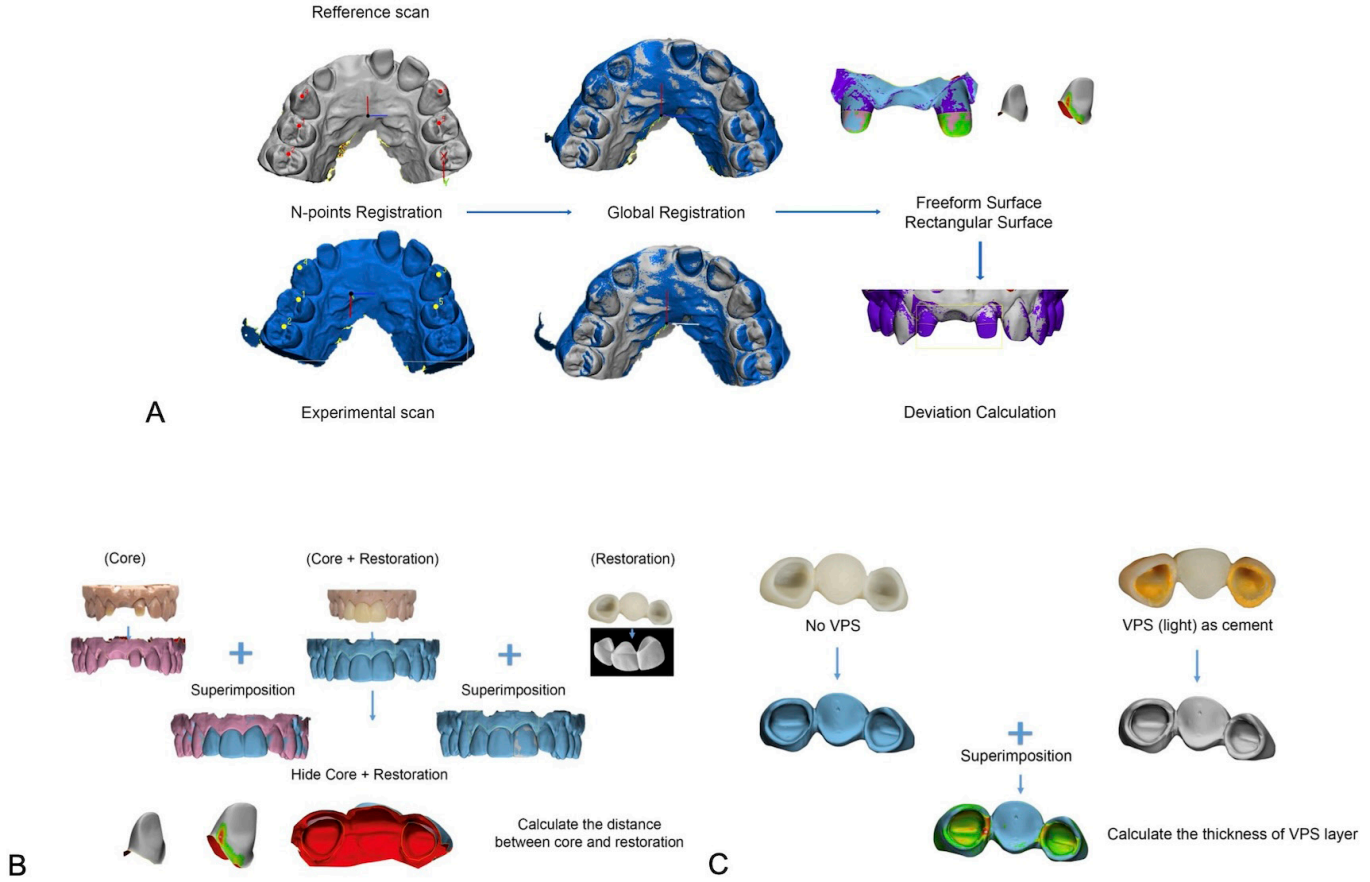
Superimposition and fitting error checks. A, Superimposition procedure. B, Fitting error check by calculation of gaps between cores and restorations. C, Fitting error check by calculation of the thickness of VPS layer.

For 3D superimposition and measurement, 3-Matic Research (version 21.0, Materialise N.V.,) was utilized. A two-step alignment procedure was used to superimpose each pair of scan data (Fig. 2a). First, three reference points were used for N-points registration: one at the incisive papilla and two at the canine tips. Next, global registration was used to guarantee that there was as little space as possible between the two models. After the superimposition was finished, the part comparison was run to display the difference between the two scans. Only in the region of abutments were the analysis results reported. The 3D image displayed the deviation result along with a color scale, where the blue area represents the outward or positive deviation of the IOS scan, and the red area represents the negative or inward deviation. Each IOS scan was superimposed with the reference scan (n=10) to calculate the trueness. Each IOS scan file was aligned with other IOS scans in the same group (n=45) to compute the precision. These parameters were noted: Mean (µm): the average distance in 3D pictures of two scans; max (µm): the greatest variance; Mean (0.05, max) (µm): A 0.05mm (50µm) criterion indicates an unacceptable mean deviation; Distribution ratio of unaccepted/total elements (%): percentage of unaccepted locations relative to total deviating locations.

5. CAD/CAM bridge restoration production and fitting error analysis.

Two ideal experimental scans from two groups of composites were chosen to design the bridge by Exocad (version 3.1, Exocad Rijeka, Align Technology). All of the steps of designing restoration were done by a well-trained CAD/CAM technician, the cement gap was set as 0.05mm, other Figures were set as default. The CAD bridges were milled using a five-axis milling machine with an accuracy of ±20 µm DWX-52D (DGSHAPE Corp) with poly methyl methacrylate resin material. The final restorations were finished and polished according to the manufacturer’s procedure.

The internal fitting of restorations was observed and evaluated using Rainbow™ CT (Dentium Co.). The CBCT measurements were then collected and analyzed using Materialise Mimic Research (version 21.0, Materialize N.V.,).

We performed fitting error check by calculation of gaps between cores and restorations (Fig. 2c). Cores only, cores with restorations and restorations only were scanned separately and then superimposed. The scanning image of core with restoration was removed to calculate the gaps between cores and restorations. We also checked the fitting error by using low viscosity Polyvinylsiloxane Impression Materials (PVS) (Fig. 2c) to simulate the cement material. The restorations were adhered to the isolated cores using PVS then removed. The models with composite abutments and restorations with PVS were scanned separately and then superimposed. We calculated the thickness of internal gaps (PVS) to determine the fitting error.

6. Statistical analysis

JASP (version 0.17, University of Amsterdam) was used to conduct statistical analysis. The data were shown as median and interquartile, or as mean ± standard deviation (SD). The Shapiro-Wilk test was used to verify normality, while Levene’s test was used to verify variance equality. Student’s t-test was used to compare independent data that had a normal distribution. The nonparametric Mann-Whitney test was used to compare data that had non-normal distributions. *P*-values less than 0.05 were regarded as statistically significant.

## Results

1. Effect of core build-up resin composite translucency on the trueness of IOS scan data

**Figure 3:**
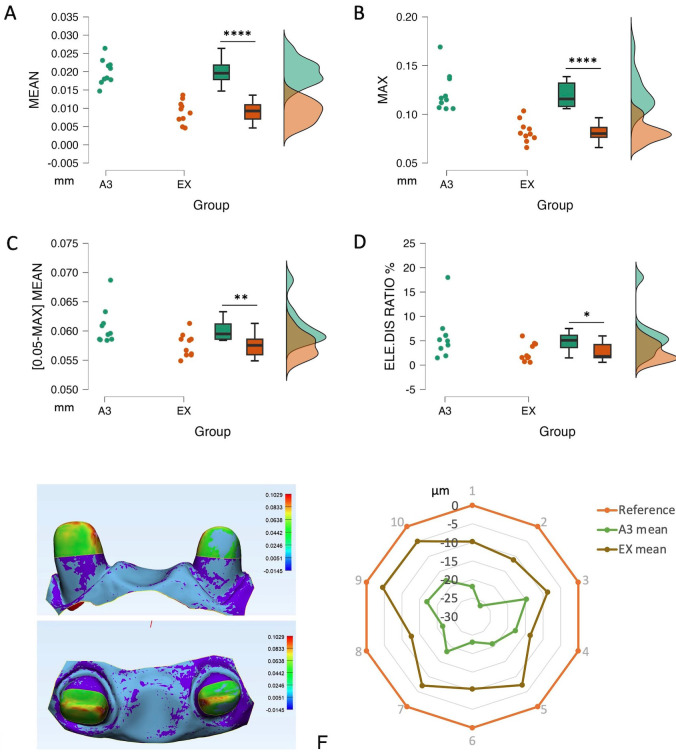
The trueness of IOS affected by resin composite translucency. (a) Mean and (b) Deviations of 2 groups of composites. (c) Mean deviations and (d) Ratio of point locating out of the acceptable threshold of 2 composites considering the cut-off value of deviations as 50μm for clinical acceptability. Data presented by dots, median and 75th-25th percentile and histogram; * *P*<0.05, ** *P*<0.01, **** *P*<0.0001. (e) The result of superimposition reference scan and experimental scan data of composite A3, deviation ladder in mm. (f) Two composite core groups had a scaled-down image compared to the reference data. Lines were presented as the mean of deviation of each group.

After 10 times of registration in both groups to the respective reference data, the results indicated that there is a scale deviation of IOS data toward the references. The results showed that the core build-up scan data from EX composite had higher trueness value than the one from A3 composite. More specifically, mean and max distortion was higher in A3 group (20 and 120µm respectively), and EX (9 and 80µm respectively) (Fig. 3a,b respectively). Considering the deviations over 50μm as clinical unacceptability, the mean deviation of (0.05-max) and the percentage of the unaccepted deviated area were collected and analyzed. The analysis elucidated that A3 composite created a higher deviation in the range of clinical unacceptability than EX composite. The percentage of unaccepTable areas over the total surface showed that almost 6% of the area of A3 data deviated further to the acceptable range, while this value in EX was around 2% (Fig. 3c,d).

The registration results were illustrated by a color scale. In the A3 composite groups, the deviation area stayed mostly on the incisal edge, mesial and distal ridge of the abutment (Fig. 3e). The trueness results of both groups were shown by the chart below (Fig. 3f). The orange outline represented the reference data whereas the experimental data included the brown line (EX composite) and the green line (A3 composite). The more centered the experimental lines are, the higher deviation is. The brown line (EX) stayed closest to the referenced line also meant that EX had the better trueness.

2. Effect of core build-up resin composite translucency on the precision of IOS scan data 

**Figure 4:**
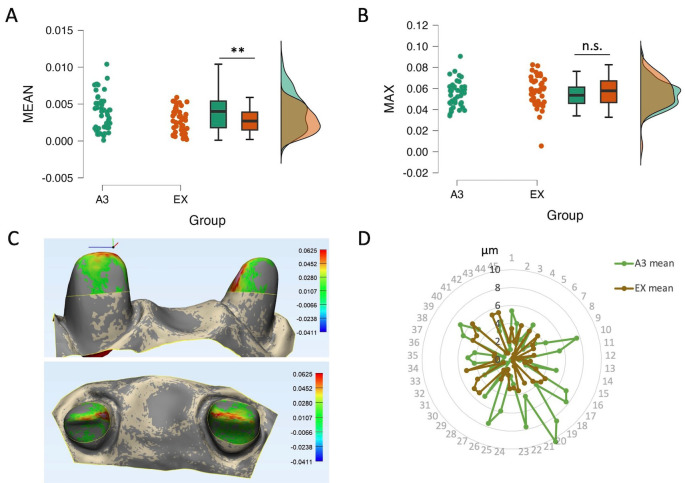
The precision of IOS affected by resin composite translucency. (a) Mean and (b) Max deviations of 2 composites. (c) The difference between 2 scans of A3 group (deviation ladder in mm) by superimposition. (d) The circle lines represented the precision of the scan data between groups. Lines presented as the mean of deviation of each group. Data (a, b) were presented by dots, median and 75th-25th percentile and histogram; ** *P*<0.01, n.s. nonsignificant.

In general, the precision of IOS scan data was higher in EX than A3 groups. Means of deviation were 4µm in A3 and 3µm for EX (Fig. 4a). There was no significant difference in max of deviation (Fig. 4b). Meanwhile, composite A3 which has higher translucency tended to create reduction especially in the incisal edge (Fig. 4c). Looking at the dispersion of the precision values, we can see that the scan data of EX is more homogeneous than A3 group (Fig. 4d). These results suggested that composite EX had the best performance in terms of precision.

3. The CAD/CAM bridge restorations resulted in fitting errors in the A3 group.

**Figure 5:**
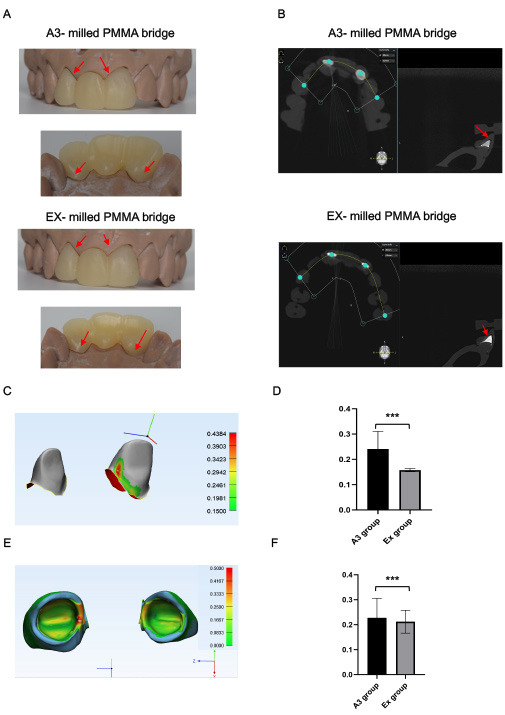
CAD/CAM bridge restorations resulted in fitting error in A3 group. (a) The A3-milled PMMA bridge showed bigger marginal gap with abutments than EX (red arrows). (b) The gap between milled-PMMA bridge and abutments of A3 & EX on CBCT file (red arrows). (c) The distance between core and restoration (internal gap) presented by a color scale. (d) A3 group showed a higher mean of internal gap (240µm) than Ex group (157µm). (e) The thickness of VPS layer (internal gap) was presented by a color scale. (f) Significant difference of over clinical acceptable threshold (150 µm) between two groups A3 (227µm). and Ex (212µm).

We produced bridges of one experiment scan for each A3 and EX groups by milled PMMA and checked the fitting to core build-up abutments. The group of composite A3 showed a bigger marginal gap than the composite EX (Fig. 5a). These fitting results were observed more clearly on the CBCT view, the bridge manufactured from the groups of composite EX performed a better margin adaptation than the composite A3 (Fig. 5b).

After registration the abutments scan with and without the PMMA bridge, the distance between abutments and bridge was calculated and analyzed (Fig. 5c). The mean internal gap was seen higher on the abutment built up with A3 composite group (240µm), which was 157µm for EX composite group (Fig. 5d). Considering 150um as the standard threshold of cement gap, we calculated the area that shot over 150µm as the clinically unacceptable zone. There was a significant difference in terms of percentage of unaccepTable zone in A3 group (5,38%) compared to EX group (0,82%).

With the use of PVS materials as the cement layer, the thickness of PVS layer (internal gap) was calculated by superimposing the restoration scan with and without abutment (Fig. 5e). A3 group showed a higher internal gap (227µm) than Ex group (212µm) (Fig. 5f). There was a significant difference in terms of percentage of unaccepTable zone in A3 group (10.3%) compared to EX group (4.8%), (Tables  Table 1, Table 2, Table 3).

## Discussion

1. Accuracy

This study presented novel approaches, including the use of composite injection techniques for the core build-up for bridge and the application of full digital workflow (IOS scanning –CAD–CAM) in restorative dentistry. Regarding the trueness, our results showed that IOS (3Shape Trios) ‘s trueness was 10-20 µm. Although we used 2 composite groups of different translucencies, Trios provided as high trueness value as other IOS devices (PrimeScan and Trios 4) (20-50 µm) (10,11). However, these studies did not show the maximum deviation (here was about 150µm), which is also an important value. The translucency of resin composite not only affects the trueness value, but also the surface noise - reflected in the irregular scan data surface and the appearance of the enlarged points compared to the real surface. The results showed that the higher translucency of the composite, the more noise there is. Deviation or noise was located more frequently in the incisal edge or line angles, where the composite is thinner, and the light is more easily reflected. The scattering and reflection characteristics of the composite might be the reason for the distorted scan image. Interestingly, it is known that deviations or artifacts occurring at the incisal edge in the anterior tooth area were a big challenge for all scanners including confocal technology (12). It was explained that the incisal edges provide limited geometric characteristics for scanning.(13) Here we found that the translucency may contribute to the discrepancy in scanning data. It requires appropriate preparation design and restorative material before optical scanning.

Precision value represents the repeatability of scan data. The smaller and more converged the precision mean value, the more dependable the data. Similar to previous studies, Trios-3 performed high precision (5-10µm) as well as PrimeScan and Trios 4 (10-11µm) (10,11). Taking the translucency of composite materials into account, we have precision in descending order as EX, then A3. In general, IOS accuracy was the best performance with the EX-composite which is suiTable to core build-up for bridge restoration. 

2. Fitting error

Data acquisition by the optical impression is the initial step that determines the success of the rest procedure (14). Different studies reported the accepTable marginal gaps between the abutment and fixed prosthesis within a range of 50 to 150 µm to avoid secondary caries, gingivitis, and cement dissolution (15-18). However, the internal fitness and marginal gap are affected by many factors including the accuracy of the surface digitization, the design, and the manufacturing process. In fact, the accuracy of scan data is just one of the causes (16,19). To minimize this gap, the scanning data should be as accurate as possible. For that reason, we considered using the cut-off value of 50µm to ensure the highest standard fitting for the final restoration. Interestingly, our results indicated that A3 groups violated the 50µm limits of clinical acceptability, while EX data showed less deviation. The errors could be accumulated during the digital workflow from impression to designing, milling, and then finishing of a crown.

With the development of digital dentistry, the need for making high-quality indirect restorations is increasingly necessary than ever. The cement gap is a key factor to consider when evaluating the quality of dental restorations. It is an essential factor in ensuring the longevity and effectiveness of the restoration. There are several methods used to measure and evaluate the cement gap in dental restorations. One such method is the use of microcomputed tomography (micro-CT), (20,21) to obtain high-resolution images of the restoration and the surrounding tooth structure (20,22). Other methods such as scanning electron microscopy (SEM),(23) confocal microscopy, optical coherence tomography (OCT),(24) and digital radiography (25). Each of these methods has its own advantages and disadvantages. Taking advance of the high accuracy 3D images from IOS, study has proved the marginal gap calculation of dental ceramic restoration using 3D registration software (26).

Unfitted restorations also raise the risk of recurrent caries or accumulation of debris and plaque with subsequent gingival and periodontal inflammation (27,28). We investigated the total possible errors of dental digital workflow for CAD/CAM bridge with composite core-build-up abutments to find out what kind of composite should be used together with the Intra-oral scanner. Moreover, we also suggested that appropriate compensation should be applied when designing CAD/CAM restoration in such a situation to achieve the best results.

3. Limitations

This is the first study to evaluate the cement gap affected by the translucency of composite on the cores build-up model to the PMMA bridge. Further research is required to accurately evaluate the cement gap of ceramic CAD/CAM crown/bridge and comparison among different methods of calculating the internal gap of fixed restoration.

4. Summary

The accuracy of optical impressions can be affected by the translucency of the composite material used, causing the fitting error of CAD/CAM prosthesis. The more translucent the composite, the less accurate the impression. This means that proper compensation should be made during prosthesis design to achieve optimal clinical results. The use of low translucency reconstructive materials helps to reduce the errors of IOS, and at the same time, appropriate compensation should be used when designing restorations to provide the most accurate results.

## Figures and Tables

**Table 1:** Trueness values of separate groups. In this table, Mean: mean deviation between two 3D images; Max: maximum deviation; Mean [0.05, max]: Mean deviation unacceptably with 0.05mm (50µm) threshold; Unacceptable/Total elements distribution ratio (%): % of unaccepted locations in total deviated locations; Mean [min, 0]: Negative mean deviations; Mean [0, max]: Positive mean deviations. Data was presented as mean ± SD for normal distributions or median [75th-25th percentile] for non-normal distributions.

Group (n=10)	Mean (µm)	Max (µm)	Mean [0.05, max] (µm)	Unacceptable/Total elements distribution ratio (%)
A3	20 ±3.43	120 ±20	60 ±3.21	5.89 ±4.65
Ex	9.01 ±3.08	80 ±10	60 ±1.98	2.66 ±1.85

**Table 2:** Precision values of separate groups. Mean: Mean deviation between two 3D images; Max: Maximum deviation. Data were presented as median (75th-25th percentile) due to non-normal distributions.

Group (n=45)	AE	A3
Mean (μm)	3.92 ±2.49	2.78 ±1.61
Maximum (μm)	50 [50 - 60]	60 [44.1 - 60.25]

**Table 3:** Internal gap values of separate groups. Mean: Mean internal gap between abutments and bridge; Unacceptable/Total elements distribution ratio (%): % of unaccepted locations in total deviated locations.

Test	Groups	Mean (μm)	Unacceptable/Total elements distribution ratio (%)
VPS layer thickness	A3	227 ±78.6	10.38
	EX	212 ±45.7	4.86
Internal gap	A3	240 ±70.3	5.38
	EX	157 ±6.3	0.8

## Data Availability

The datasets used and/or analyzed during the current study are available from the corresponding author.
